# Supporting cognition in systems biology analysis: findings on users' processes and design implications

**DOI:** 10.1186/1747-5333-4-2

**Published:** 2009-02-13

**Authors:** Barbara Mirel

**Affiliations:** 1 School of Education, University of Michigan, 610 East University Avenue, Ann Arbor, Michigan, USA

## Abstract

**Background:**

Current usability studies of bioinformatics tools suggest that tools for exploratory analysis support some tasks related to finding relationships of interest but not the deep causal insights necessary for formulating plausible and credible hypotheses. To better understand design requirements for gaining these causal insights in systems biology analyses a longitudinal field study of 15 biomedical researchers was conducted. Researchers interacted with the same protein-protein interaction tools to discover possible disease mechanisms for further experimentation.

**Results:**

Findings reveal patterns in scientists' exploratory and explanatory analysis and reveal that tools positively supported a number of well-structured query and analysis tasks. But for several of scientists' more complex, higher order ways of knowing and reasoning the tools did not offer adequate support. Results show that for a better fit with scientists' cognition for exploratory analysis systems biology tools need to better match scientists' processes for validating, for making a transition from classification to model-based reasoning, and for engaging in causal mental modelling.

**Conclusion:**

As the next great frontier in bioinformatics usability, tool designs for exploratory systems biology analysis need to move beyond the successes already achieved in supporting formulaic query and analysis tasks and now reduce current mismatches with several of scientists' higher order analytical practices. The implications of results for tool designs are discussed.

## Background

Technological advances have made exploratory analysis in systems biology accessible to large numbers of "everyday" biomedical researchers – i.e. researchers who conduct analysis without the help of customized tools or specialized collaborations with bioinformatics experts. Yet, as usability studies show, initial query and analysis tasks that bioinformatics tools now make possible for scientists are not coupled with the support scientists need for generating deep insights for hypothesizing. Deep insights as used here refer to new knowledge scientists derive from interpretations and inferences about sets of biological relationships, changes, and effects that explain putative disease mechanisms [1]. Cohen and Hersh suggest that this problem of insufficient support may be traced to bioinformatics tools inadequately fitting researchers' actual exploratory practices [2]. To design for a better fit, Cohen and Hersh argue, bioinformatics specialists need to conduct more naturalistic investigations of biomedical researchers at work on real world analyses. To fill this need, I conducted field studies of 15 biomedical researchers as they conducted their actual systems biology analysis and aimed to formulate some hypothesis about mechanisms of a complex disease. All the scientists interacted with the same web-based protein-protein interaction application, an application that supports query and analysis and that includes a visualization workspace.

The goals of the field study were to examine whether support for generating deep insights was adequate and, if not, to analyze whether inadequacies were due to a mismatch between tools and scientists' actual analytical processes and strategies. The field study sought to uncover what these higher level cognitive processes are and how they need to be better supported. Findings reveal that better support for deep insights and hypothesizing is needed, and findings raise the open design question of whether support for all aspects of scientists' flow of analysis can or should be addressed in one tool or many. Regardless of design decisions about this issue of scope, the field study traces the problem of inadequate support to three mismatches between tools and scientists' higher order cognitive processes. Specifically, mismatches occur for the core discovery-driven analysis processes of: (1) validating evolving analyses, based on domain-driven standards of excellence; (2) making a transition from classifying relationships to causal modelling to explain biological events and outcomes; and (3) creating viable and credible causal mental models that can lead to defensible hypothetical biological stories. Field study results suggest that for the tool versions tested – and for the many other tools like them available to scientists – systems biology applications are at a critical juncture. Tools have advanced to the point of being able to support users fairly successfully in finding and reading off data (e.g. to classify and find multidimensional relationships of interest) but not in being able to interactively explore these complex relationships in context to infer causal explanations and build convincing biological stories amid uncertainty.

In bioinformatics, requirements for meeting these objectives are still underspecified. Consequently, it is difficult to determine if one or many tools are needed to meet scientists' ways of knowing and reasoning for explanatory analysis under uncertainty. Findings from this study help to guide such requirements, providing an empirical basis for deciding optimal scopes and conceptual designs.

The rest of this article reviews relevant literature, describes field study results, and critically discusses mismatches between tools and cognition. Mismatches are tied to design issues, many of which the current research literature acknowledges but underspecifies. The conclusions propose objectives to guide bioinformatics specialists in developing conceptual design requirements. Methods follow. Additional file 1 proposes in more detail design strategies to guide the specifications of requirements for conceptual designs aimed at a better fitness to purpose and greater usefulness.

## Review of relevant research

Several bioinformatics usability studies overtly or tacitly establish that scientists are not able to gain deep insights from bioinformatics tools intended to support exploratory analysis. In a 2005 study [3], 30 scientists explored microarray data with five different tools but could only attain surface level insights. They gained surface insights by directly reading off data from interface displays, attaining, for example, new knowledge about structural and functional traits of genes, clusters with similar properties, and expression values that were statistically significant. Deeper inferences eluded scientists, for example, insights into roles played by functional and structural relationships in chains of interactions and effects on disease processes. Other bioinformatics studies – involving both naturalistic and simulated tasks and interviews – reveal the same problem [4-10].

Unfortunately, little if any research in bioinformatics directly analyzes scientists' processes for and impasses in gaining explanatory insights amid uncertainty from a cognitive perspective. But research exists in other disciplines that focuses explicitly on scientists' cognition, and it can lay a foundation in bioinformatics for understanding biomedical researchers' higher order ways of knowing and reasoning in systems biology. Many researchers converge on the same cognitive characterizations. Neressian, for example, finds that regardless of specialty when scientists conduct discovery-driven investigations they progressively and, at times, recursively engage in three modes of reasoning: Classification, model-based reasoning, and narrative reasoning [11]. Other studies find the same combination and integration of reasoning processes [12-15]. As cognitive researchers argue, none of these modes of reasoning alone is sufficient for hypothesizing; all must interact coherently and dialectically [16]. Furthermore, in each mode scientists continuously alternate between creative hunches ("loose analysis") and validation ("strict analysis) [17].

In their studies, bioinformatics researchers indirectly help to expand on one of these reasoning modes – classification. Though conducted for purposes other than design, a handful of bioinformatics studies strive to model tasks and protocols for query and analysis. For example, bioinformatics researchers find that bioinformatics specialists functionally analyze gene sequences by differentiating relationships based on such categories as conservation, sequence similarities, diseases, and biological structures and functions [5,7,9]. Correspondingly, these specialists' flows of classification typically involve collecting objects into categories, filtering and restricting them, and subjecting them to conditional analysis [7]. Some of the usability studies discussed previously show that bioinformatics tools typically support this reasoning to a certain degree, hence the achievement of surface insights. Unfortunately, classification alone does not lead to hypotheses that are novel, credible and plausible. Causal mental modelling and narrative reasoning must occur and be supported by tools, and they are more difficult to formalize.

Model-based reasoning, as it functions in hypothesizing about systems biology problems, involves drawing inferences to explain how and why biological events, changes, and outcomes under various conditions and mediating constraints may influence mechanisms of a particular disease. When scientists engage in model-based reasoning they go beyond read-offs – the focus of classification – and infer contexts, conditions, and their functional and structural effects on biological interactions and outcomes [18-20]. Cognitively, inferences are the most difficult relationships to draw and validate [20].

Using multiple reasoning strategies, scientists draw causal inferences by turning structured – that is, quantitative or categorical – knowledge representations into qualitative mental models of changes and effects [21]. Bioinformatics systems cannot build in rules for this inferential thinking and causal modelling because these cognitive processes depend on scientists' domain expertise, heuristic strategies for analyzing local biological contexts, and opportunistic and emergent analytical pursuits [22].

For this cognitively demanding and complex reasoning, the match scientists need from tools is cues, not read-offs of data elements per se [14,23,24]. Unfortunately, cues are an under-specified human-computer interaction requirement in general and in bioinformatics specifically. In addition, for mental modelling scientists need to selectively discern biologically meaningful concepts and to manipulate data relationships, especially through spatial transformations [14,18]. Spatially, scientists seek to externalize such mental processes as rearranging relationships to explain causes and effects to view them from different perspectives and contexts. This need may demand aggregating data in different ways and strategically laying out and re-laying out data to discover plausible associations and effects. For example, in systems biology spatial transformations can include creating overlays of a "disease-ome" mapped to molecular interaction networks or side-by-side views for comparison [25].

Finally, scientists engage in narrative reasoning, which evolves from and refers back to mental modelling and classification. As Latour emphasizes, scientists continuously build stories to understand causal relationships. He writes, "Most of a scientist's ingenuity goes into designing devious plots and careful staging to make the actant participate in new and unexpected situations that will actively define it" [15]. This effort is not trivial, and, in fact, is elusive in the support most current bioinformatics software offers. Only a handful of applications have ventured into trying to provide such support, and the tool versions in my field study did not strive to do so [26,27].

For systems biology, these complementary and integrated modes of reasoning are directed toward uncovering causes and effects in chains, networks, and loops of interacting biological entities and processes. For such complexity, analysis workspaces that include interactive visualizations are central to tool designs. Visualizations are "the equivalent of power tools for analytical reasoning." Many well-documented visual design guidelines exist but they pertain to low level visual analysis tasks and operations, not to the higher order cognition that is the focus of this field study [e.g. [28-30]]. Some visual analytics researchers in bioinformatics and other domains highlight this gap and argue that for complex analysis users need better visualization support [31,32]. For example, users need support for representing and analyzing weighted relationships and outcomes, clarifying possible sources of causation, and discovering co-occurring relationships and constraints [31].

Various findings related to interface and interactive visualization designs for complex analysis have important implications for systems biology but only insofar as the designs can be adapted to scientists' actual practices and purposes. Results from my field study on scientists' ways of knowing and reasoning for real world purposes in systems biology provide guidance for adapting design strategies for complex analysis to the demands of systems biology.

## The tools

Fifteen scientists in the field study all interacted with the same web-based tool, which gave them access to the Michigan Molecular Integrating (MiMI) protein interaction database http://www.mimi.ncibi.org. MiMI is an innovative database that uses advanced computational merging, integration and provenance tracking to bring together the content of (at the time of the testing) ten well-established protein interaction databases. Additionally, MiMI integrates and stores conceptual information about the molecules, such as Gene Ontology (GO) annotations and articles referencing specific molecules or protein interactions. MiMI is also the name of the web-based, front-end query and analysis tool for the database. Both the database and the tool are developed and maintained by the National Center for Integrative Biomedical Informatics (NCIBI), one of seven NIH-funded centers dedicated to generating and disseminating advances in translational bioinformatics. Informal comparative assessments between MiMI and other comparable innovative open source tools are regularly conducted by NCIBI developers and consistently show that MiMI is representative of and in many ways better than tools available today to users. Thus results from the field cases likely reflect the state of bioinformatics tools today in supporting explanatory and exploratory analysis. Few tools today explicitly set their scopes specifically on supporting only one, all, or some of the multiple modes of reasoning on which formulating hypotheses in systems biology depends. Largely, little is known about such multi-mode reasoning for explanatory analysis in systems biology. Thus the trend in tools seems to be to provide information, see what users do with it, what more they want to do, and modify the tools accordingly.

MiMI actually offers several exploratory capabilities beyond "typical" systems. For example, using MiMI's web interfaces to the database, scientists are able to gain access to large volumes of heterogeneous data in one place and to retrieve, as well, details derived from the mining of PubMed literature through semantic natural language processing (NLP) distinct to the MiMI suite of tools. MiMI also lets users link out to other available knowledge sources on the web.

Another strength of MiMI is that it offers a plug-in to Cytoscape, which carries MiMI data over to Cytoscape and displays the data in dynamically linked tabular displays and networks of protein-protein interactions. Cytoscape is a highly cited, award-winning interactive visualization system for biomedical research http://cytoscape.org. The MiMI plug-in for Cytoscape (referred to here as MiMI-Cytoscape) provides the same link-outs found in MiMI along with links to extracts from articles mined through NLP, and a link to a MiMI-integrated subgraph matching tool called SAGA. SAGA lets scientists find overlaps between a MiMI-Cytoscape sub-network of interest and KEGG pathways.

The MiMI and MiMI-Cytoscape tools used in the field research were beta versions. Based on feedback from the ongoing user observations and interviews, NCIBI developers steadily improved upon and sent updated versions back to the field. Using MiMI, the scientists queried the database and interactively analyzed results for insights into mechanisms of a disease or other aberrant cellular process. The scientists all accessed extensive data from the MiMI database on biological concepts such as Gene Ontology (GO) annotations or types of experiments used to find the protein interactions. With the plug-in, the scientists explored high dimensional molecular interactions and moved recursively between Cytoscape and MiMI displays.

## Results

### Overview of common research and analysis practices

Fifteen scientists researched distinct problems related to different diseases and putative mechanisms. Regardless of these differences, all 15 of the scientists commonly started with candidate genes from prior experiments and queried the MiMI database for results on gene attributes and gene product interactions. From query results, they conducted the same type of analysis. They all sought to identify interactions and genes that were previously unknown and to find links between previously unknown genes/gene products and those known to be associated with mechanisms of their targeted disease. Ideally, the scientists wanted to contextualize this interplay of known and unknown genes to discover a plausible, credible biological story about disease mechanisms, a story convincing and credible enough to report to colleagues and test through further experiments. They did not conduct comparative analyses such as uncovering connections between different diseases or associate phenotype and genotype data.

All of the scientists structured their inquiries in similar ways, and all engaged in the same higher order reasoning processes. Specifically, the scientists progressively, and at times recursively, reasoned by classifying, by attempting to build mental models of causal relationships over time and space, and by trying to put together plausible and credible biological stories. All of the scientists validated continuously and conducted their work through the same stages of exploratory analysis (see Table 1).

**Table 1:** Stages of exploratory and explanatory analysis shared across scientists

Stage (its dominant reasoning)	% completing this stage	Description
Confirmation (validation)	100%	Scientists vetted query results and the tool for accuracy, reliability, and timeliness *Example: Look for familiar literature references.*

Separating Wheat From Chaff (classification and validation)	85%	Scientists classified relationships to find genes and protein interactions of interest *Example: Locate an interaction between a candidate gene from experimental findings and a gene product known to be associated with a disease*

Beyond Read-offs (Model-based reasoning and validation)	0	Scientists wanted to place relationships of interest in local and global contexts to mentally model explanatory biological events relevant to a disease. *Example: Contextualize significant regulatory relationships in pathways*.

Story-building (narrative reasoning and validation)	0	Scientists sought to turn explanations about biological events into new, credible and plausible biological stories. *Examples considered to be credible were not available based on scientists' progress.*

Results cover only the first three stages of analysis shown in Table 1. As can be seen from the percentages of scientists who completed each stage in the table, scientists did not complete causal modelling or story building. I now turn to the knowing and reasoning behaviors that the scientists shared in each stage of their real world research.

### Cognition and analysis in Confirmation

During Confirmation, scientists primarily sought to trust the accuracy and completeness of the data and to be assured that the concepts, data, and interactivity offered by the tool fit their analytical intentions and practices as scientific experts and researchers. Scientists' levels of trust affected their immediate willingness to engage with the tool for their intended explorations and tacitly influenced their sustained engagement. These validation processes took the form of affirmation. Scientists perused results and applied the following processes and actions:

As Table 2 shows, the tools satisfied a great deal of scientists' needs for Confirmation. For some needs, partial support was the best tools offered. When support was partial, scientists often spent a good deal of time – upward of ten minutes – searching for information or navigation links that, in fact, the specific version of the tool did not yet include. The scientists reported that this amount of time for confirmation was too long. As one scientist commented, "It would be nice to see when no data are available instead of having to hunt around." The scientists may have been annoyed at this point but not enough to keep them from moving forward in analysis to classification reasoning.

**Table 2:** Confirmation stage processes of validation

Processes of Validation	Actions	Supported?
Compare results to one's own experimental findings for affirmations and contradictions	- Sort by gene name - Scroll to genes of interest - Hyperlink to details relevant to experiment	Fully

Determine accuracy of results by hunting for redundancies or synonyms	- Sort by gene name - Scroll to various known genes - Check "Other gene name" field - Scroll to assure no duplication are displayed	Fully

Determine the accuracy of the data and the data sources	- Search for cues or information indicating "data autobiography" or provenance (e.g. source database, last date updated, logic or rules for matching and integrating items across multiple databases sources and for determining a common Gene ID) - Judge the credibility of the underlying logic	Partially Not easy at the time to uncover inner processing

Determine the completeness and relevance of the data	- Sort by gene name - Scroll to find various known genes or interactions and see if they are included - Confirm expectations for: # of interactions # of literature sources for a gene, names of interactors, dates of literature references, GO annotations - If data do not confirm expectations, look for cues indicating information about logic and rules for merging and integrating data	Partially Did not at the time provide counts

### Cognition and analytical practices during Separating the Wheat from Chaff

In the next stage, Separating the Wheat from Chaff, scientists classified genes and interactions by reading off data from the displays. They aimed to winnow down the large set of query results to relationships of interest for further explanatory analysis. They read off and interpretively related multiple factors, many of which they had earlier confirmed but only in a cursory fashion, such as GO annotations for molecular function, cellular component, biological process, and homology. Now making critical and binary judgments, the scientists uniformly sought to separate "interesting" from "not interesting" genes and relationships.

Many of the scientists uncovered to their satisfaction known genes/interactions relevant to their targeted disease. For example, one researcher of bipolar disorder found interactions involving FOS, which he knew to be associated with response and non-response to lithium treatment. This finding of a known response gene triggered the scientist to examine more known and unknown interactors of FOS and later to branch out from select interactors to analyze unfamiliar and potentially meaningful functional clusters. The tool design functioned well for finding known genes and interactions.

The scientists also specifically looked for unknown genes and interactions. Rarely were they able to make these judgments simply by focusing on one property, as this scientist happened to do: "It's an RNA phenotype," he declared. "Embryonic lethal. That means it is important. It doesn't tell me more than that. It just tells me it's important." Rather the scientists mostly classified data by interrelating many factors at once drawn from several sources. They often combined their commonly applied heuristic strategy of looking for functionally similar molecules with other strategies and data, as this scientist reveals:

The Description shows this one has the potential to be involved in some of the processes we're interested in. [She links out to BLAST to find what other organisms have the protein]. This interactor is in all organisms but yeast. That's a good sign that I didn't know. It's in all the things we study. Now let's see titles on literature about it. The titles suggest this protein is involved in transcription in the nucleus, and that's unrelated to what we do. But it's kind of complicated. These proteins are thought to function in the nucleus and cytoplasm. It makes me cautious that there are all these papers citing a function in the nucleus. I'm cautious but it doesn't entirely rule this protein out for me. So I'm interested in this one very much. That's my third interesting protein so far [out of 12 that she has explored].

The scientists knew that attributes such as function can vary within a gene by time and tissue and that regulatory proteins might have multiple disparate and, at times, opposing functions depending on cellular context. Thus even during read-offs they dealt with uncertainty.

Classification processes during the Separating the Wheat from Chaff stage were often lengthy – lasting 30 minutes or more. In this process, scientists often analyzed anywhere from 20 to 50 interactors of genes of interest, a complex task, as exemplified by this biomedical researcher's comment: "The problem is that there are all these potentials, and it takes so much time to go through each one and figure out what this means." Notably none of the scientists saved or commented aloud about wanting to save anything of interest that they found here or later. Saving seemed to require more confidence from ongoing validations than the scientists had achieved.

Validation processes took a different form in this stage from the affirmations of Confirmation. Now seeking to establish reliability, the scientists judged whether interactions of interest might be false positives. Ideally, scientists wanted test statistics:

What is the p-value of this interaction with MYC? Why does MiMI think there is an interaction? Based on what literature? I need a p-value to show the likelihood of the interaction and the strongest interactions. I want listed interactions ordered by p-value.

Most of the scientists recognized that open source tools such as MiMI – tools that integrate data from public databases – were unlikely to display test statistics. As an alternative, the scientists figured they could link out to PubMed and scan relevant articles for p-values. Linking to the literature, however, was time consuming, and the scientists asked if there was a way to "filter" (meaning, construct complex queries) to see only the literature relevant to certain properties of interest. Unfortunately, an easy-to-use complex query form was not yet available, and scientists could not get to relevant literature quickly to validate the reliability of "interesting" items.

The scientists soon grew impatient with continuously clicking to various articles in seemingly random ways. They instead sought surrogate evidence for reliability, evidence they could extract directly from MiMI or MiMI-Cytoscape displays. They found and were pleased with the following surrogates for validation: Counts of peer-reviewed articles that cited an interaction or protein ("the more the better"); counts of experiments (# of evidences) producing the same outcome ("the more the better"); and types of experiments producing outcomes ("I'm skeptical of yeast 2 hybrid. It has too many false positives"). Other surrogates included extracted sentences about interactions mined from full text PubMed Central articles – a feature implemented in later versions of MiMI-Cytoscape during field testing – and scientists' reliance on their own prior knowledge, for example:

I see FLO9 interacts with a protein that I don't know. I can see where it's logical. They're functionally similar though not identical. Both have something to do with conditions of metabolism and nutrient status in the cell. I can see some connection.

Two of the scientists who did not achieve ample confidence reached an impasse due to a lack of confidence at this stage of analysis. One of them, a biostatistician, demanded test statistics. The other, a biomedical researcher who was examining members of the Bcl2, Bax and BH3 subfamilies based on two years of research, wanted to immediately identify and move quickly to literature she had not previously read. Unable to sufficiently achieve this objective, the researcher's trust in the tool's ability to address her intended analytical practices diminished. She subsequently downgraded her goal from hypothesizing about the protein family to browsing the literature for other projects. The other scientists eventually reached impasses, as well, but not until later in the flow of analysis.

### The transition from classification to mental modelling

After finding genes and interactions of interest, scientists tacitly began to shift from asking "What?" to asking "How?" and "Why?' questions for causal explanations. One of the scientists who had found relationships of interest now moved forward, describing this transition as follows:

Lists don't make it now. These genes and interactions are important for biological reasons. What is the common element they bind to? How do they interact and is that route affecting cancer progression?

For their narrowed down set of interesting genes/interactions, scientists sought to relate the set to cues and multi-dimensional information that would credibly suggest and contextualize biological dynamics of change, stability and consequence. During the transition, the scientists notably did not seek to engage in full fledged model-based reasoning yet. They simply wanted evidence that they could, given the tools and data. Specifically, scientists looked to see if the tools provided the following:

Is there a physical interaction or regulation between them? It's vital to know what kind of connection it is to know functions. Does it belong to the same family? Is it a positive or negative regulation?

Can I find possible targets where auto-antigens can be present, especially binding sites. I'm also looking for insulin receptor subunits. It would be great to see a break down of the alpha and beta chain to see if we can find more information on the receptor itself.

Filters are too limited. I need to filter on molecule type. I [also] need to know relationships between molecules other than interaction. There could be something going on that is not a protein-protein interaction or protein-DNA interaction.

As part of this initial mental shift to causal modelling, some scientists began to contextualize their interesting sets by relating networks to data on genes from their own experiments, such as significance statistics on differentially expressed genes. One scientist who was studying the efficacy of lithium in bipolar disorder manually checked network displays against his spreadsheet of expression data and commented:

If I manually cross-check this graph to some of the genes from my experiment. I see that in the Wnt pathway the transcription factor GSK3 in this sub-network of interest is down. But that doesn't make sense for lithium treatment [which inhibits GSK3]. And Beta Catenin should be activated. That should make cells grow. Everything should be up. Why is GSK3 down? Hmm. TCNF is down. Let me look at the genes around TCNF. SMAD4 is down. Let me look at the TGFb pathway where it connects to SMAD4. What genes in that pathway may be making SMAD4 go down because they are inhibiting TCNF?

This scientist and several others in the field study would have liked to import into Cytoscape his experimental statistics about genes that were significantly differentially expressed. Data import was available in Cytoscape but the scientists unfortunately did not know it was. Consequently, none brought in data. Some instead manually cross referenced their own tables with the tools but cognitively they could do very little complex multidimensional analysis and validation this way.

As the scientists made a transition from classification to causal mental modelling, they also wanted to be sure that items they had deemed interesting so far – and worthy of additional analysis – were, in fact, interesting and worth the effort. They validated by looking closely at links connecting proteins (nodes) to see if it was possible with the tools to differentiate types of interaction (regulatory, physical and the like) or types of experiment showing an interaction. Additionally, they sought to validate whether network configurations involving their interesting genes and relationships were by chance alone, as seen in the following think-alouds:

Is there even something significant about this network structure that would not occur just by chance?"

I fear making a connection between these genes and [the] Wnt [pathway] just because they are related at 3 steps out or less. If I randomly give two gene names, how many genes are there between by chance alone?

When pressed to describe a model that might fit these desired tests of significance, however, the scientists were uncertain. They all said that because they sought confidence in the biological significance of a network, they did not merely want common network statistics, such as hypergeometric distributions or average connectivity. Unfortunately, for this hard problem of singling out biologically meaningful network structures, none of the scientists could specify the relationships that might suffice for a statistically sound model, and few of them realized the nontrivial computational demands this significance testing implied. Notably, no scientist sought predictive measures, perhaps due to an inadequate foundation of confidence at the outset.

Table 3 details the evidence and available interactivity the scientists sought.

**Table 3:** Common knowledge representations desired the transition

Analytical Function	Knowledge representations/interactions needed for the transition to causal modeling	Available?	Easy to view/anticipate using?
Contextualizing	Expression values from one's own experimental data as cues about regulatory processes and paths	✓	Yes, but not found
	Indirect relationships and paths between proteins in networks of interactions	✓	Required time-consuming filtering
	Functional pathways related to sets of interactions	✓	SAGA, but not inter-active; or side by side
	Homology or pathway comparisons between species to see if molecules or interactions are conserved	✓	Yes
	Ability to filter by multiple variables at once or to filter to only the shared interactors between specified genes	No	No; strings in a field
	Test statistics	No	No

Detail	Types of interactions: Physical binding, activation/inhibition, family member interactions, transcription/expression, translocation/secretion, phosphorylation	Partly	Buried in NLP free text; incomplete knowledge
	Types of molecules: Distinctions between genes, proteins, chemical effectors DNA, mRNA, protein complexes, mRNA, enzyme	Partly	Incomplete knowledge
	Experiment type	✓	Strings in a field
	Ability to color code by attributes	✓	Time consuming

As Table 3 suggests, the knowledge representations that the scientists required for making a transition from classification reasoning to causal modelling ran the gamut from straightforward usability improvements (e.g. a greater transparency in available data import features) to uncharted computations (e.g. test statistics). Table 3 also shows that development efforts for many of these user needs are non-trivial (e.g. embedding adequate interactivity into SAGA and KEGG pathway graphs and adding ways for users to manipulate views and data). For this set of wide-ranging user needs, the scientists' analytical demands were not adequately satisfied in the versions of the tools used in the study, and only three of the 13 scientists continued to the Beyond Read-offs stage for deeper insights.

### Cognition and analytical practices during Beyond Read-offs

The three scientists who engaged in Beyond Read-offs did not get very far in this stage. Thus results in this section depend more on interview responses than in previous sections. During interviews the scientists discussed insights they would like to have gained in the Beyond Read-offs stage, framed as questions and covering the following relationships:

• How do relations between biological entities change when the same genes are considered in different functional contexts?

• How do protein structures and composition change based on cellular conditions?

• What integrated and coordinated functions occur in complexes and relationships between them?

• What regulatory processes, agents, and intermediating conditions are at play that may explain the disease being studied?

Conceptually and technologically, this reasoning required more immersion into biological uncertainty and incomplete knowledge than analysis so far had involved, and it required more deliberate inferential reasoning. Inferences included the use of multiple strategies and reasoning across scales. To draw inferences, scientists sought cues about localized biological events – conditions, contexts, and interactions that potentially influenced biological functional behaviors, changes, and self-regulation. For example, the scientists coupled analysis of protein interactions and pathway networks to see if this cross referencing might suggest mediating groups of genes for a given disease. For these questions, the scientists went beyond read offs and, in doing so, expected to interact with the data with a great deal of user control.

The scientists became stymied when interface displays did not afford adequate interactivity. For example, referring back to Table 3, tabular displays that were linked to interactive network displays let scientists read off many important pieces of content, such as interaction type, molecule type, experiment type, and concatenated strings of canonical pathways and GO annotations. Notably, multiple attributes pertaining to a gene were formatted as concatenated strings, and some fields themselves were defined as a compilation of many values – e.g. "Interaction Type" contained values for interaction type, experiment type, *and *biochemical reactions. For Beyond Read-off purposes, however, this data structuring obstructed the scientists from grouping and selecting items based on single attributes and from seeing just the selected items displayed and highlighted in the network view. Nor could the scientists color code or aggregate by individual (not stringed) field values. Additionally, with this version of Cytoscape and with plugs ins other than MiMI available at the time, the scientists could not selectively extract sub-networks by trait (e.g. pathway) and further manipulate or contextualize just the sub-networks. As one of the three scientists participating in Beyond Read-offs commented in think-alouds:

These genes are involved in multiple concepts. Now I need to look at them biologically and single out just this one annotation. Is this concept up in the context of a cell that is "just resting" but down in a cell that is proliferative?

Causal inferences, moreover, had to be warranted and defensible. The scientists who engaged in Beyond Read-offs looked once more for statistical evidence that network structures had potential biological meaning and were not simply chance occurrences. For example, when the scientists used SAGA within MiMI-Cytoscape to match potentially meaningful sub-graphs embedded in the whole to canonical pathways, they wanted to know how confident they should be in the match, given the accuracy of the underlying similarity matching algorithm. The scientists also wanted to link to and revisit literature about relationships of interest to read details that would help in judging credibility.

In the field study, scientists spoke about the content, available interactivity, and workspaces they would liked to have had to actualize their beyond read-off intentions (See Table 4). Some of these overlapped with transition stage needs:

**Table 4:** Support scientists would have liked for explanatory analysis

Content	Edges in networks weighted by biological traits
	Overlays of protein-protein interactions and disease associations
	Overlays of protein-protein interactions and relevant pathways
	Distinctions between proteins and other molecules that might serve as mediators of interactions, e.g. enzymes
	Test statistics and counts (e.g. # of interactions, # of articles, overrepresentation of a functional term) and perceptually encoding nodes or links by them
**Interactivity**	Updating of interactions (e.g. selection, color coding) across views – e.g. across overlaid networks
	Facile filtering (users had to use mini-scripting to filter)
	Facile color-coding (at the time it took 15+ steps to color code)
	Integrating one's own data into the displayed dataset
	Simplifying networks through zooming, filtering, color-coding, expanding some nodes but not others, mapping only select neighbors to pathways
	Conducting computations on networks to find e.g. shared paths to identify indirect interactions or recurrent or aberrant patterns that might signal a biologically significant set of relationships

**Workspaces**	Spaces for comparing different networks side-by-side with dynamically linked interactions
	Spaces for aggregating entities on the fly into manipulable qualitative attributes based on emerging knowledge (e.g. normal vs disease conditions)

Unfortunately, with these versions of the tools, none of the scientists found enough of what they were looking for, and none was able yet to gain deep insights for building hypothetical stories.

## Discussion

Results show many common patterns across cases in scientists' ways of knowing and reasoning but suggest, as well, that creating a match between a tool and a scientist's cognition might be specific to a scientist's domain specialization and analytical objectives. The biostatistician, for example, as a statistics specialist distinctively cut short even her surface level insights for want of hard test statistics. The biomedical researcher, whose prime analytical objective during the Separating the Wheat from Chaff stage was to quickly hone in on relevant and previously unknown literature about her target gene family, also encountered impasses but in her case because of her distinct analytical objectives.

In the rest of the field cases, results show that MiMI and MiMi-Cytoscape, which are emblematic of other bioinformatics tools, have advanced to enabling scientists to access and understand rich stores of heterogeneous data. The tools provided important content and displays that facilitated scientists in well-structured or formulaic aspects of query and analysis tasks involving reading off data to confirm query results, locating known and unknown genes/interactions, and separating out those of interest. The scientists were able to make critical judgments and learn new things from the data. Moreover, these tools made major steps in providing surrogates for test statistics to help scientists achieve confidence. These advances have opened the realm of systems biology to countless everyday scientists in biomedicine. But results also suggest that in making these tasks accessible the tools sparked scientists' motivations to go beyond read-offs. As results reveal, designs beyond read-offs have to differ in kind not just degree from designs for straightforward data extraction for affirmations or classifications.

Admittedly, analytical integration for hypothesizing is a long exploratory process, and it may not be surprising or troubling that scientists did not achieve a well-developed hypothesis at the close of the observation periods. But findings from this study – and from others – suggest that tools today are not sufficiently launching scientists on this long term hypothesizing process to begin with. This whole process requires *integrating *classification, mental modelling and narrative reasoning – that is, facilitating the move from lists to stories. Whether bioinformatics developers strive to build one tool or many to fulfil this purpose, the goal must be to support scientists in integrating complementary modes of reasoning and in interweaving "loose" and "strict" analyses for explanatory inquiries under uncertainty. Supporting only one mode of reasoning isolated from the rest – regardless of which mode it is – or insufficiently supporting transitions between them impedes the formulation of hypotheses.

One thing that is clear from the results in the Transition and Beyond Read-off stages is that "information delivery" is not enough to satisfactorily support this analytical flow and integration. In the field study, a good deal of the data relevant to scientists' transitional thinking and causal mental modelling was available ("deliverable") from the tools but was often hard to find or time-consuming to encode into graphics. Equally if not more important, available data were not represented as scientists needed them to be for the intellectual processes of mentally modelling causes and outcomes. For example, the data were not represented in ways that conveyed or cued biological meaning and credibility. Moreover, for the manipulations of data that are prerequisite to constructing knowledge during causal mental modelling, the data often were not structured right, for example for users to cluster, filter, color code, and lay out graphs. As researchers in visual analytics show, different types of analysis tasks demand specific visual representations of knowledge. For example, users benefit most from side-by-side views – such as the network and tabular views in MiMI-Cytoscape – when their tasks involve detecting patterns of interest and making transitions to new modes of reasoning. But they need single views rich in relevant information and conceptual associations when their goal is to understand causal relationships and diagnose problems [33]. Conceiving and then designing these rich views are vital but challenging.

The scientists in the study did not engage seriously in narrative reasoning but research suggests that support for story building also differs dramatically from support for classification reasoning and read-offs. In story building, scientists use narrative structure to recount chronological events and tensions leading to an explanatory result, events with actors, actions, scenes/contexts, themes, and agency. For example, they may associate and synthesize their cumulatively generated knowledge to tell an hypothesized story about two potentially influential proteins in a localized context – the star actors and scene in the story. The story would capture how the proteins are encoded by genes that lie on separate disease-relevant chromosomes, how they function in a disease-related intracellular signaling pathway, and how evidence suggests a genetic variant that may cause one of the proteins to vary in structure, activation, and localization, thereby affecting variation in the other protein in disease susceptibility. Noting the challenge of supporting this mode of synthesizing knowledge into convincing stories Kumar et al. remark that for scientists story building can actually become inhibited not helped by data or "descriptor overload," a phenomenon that occurs when displays "deliver information" but fail to draw scientists' attention to salient relationships and cues about specific biological contexts, conditions, events, and actors, and interdependent actions [26]. Similarly, research shows that overly-formalized narrative objects and their properties do not effectively support narrative reasoning [27].

My field study results show that for the explorations scientists conduct in explanatory systems biology analyses, tool designs and scientists' cognition are mismatched in the following three ways:

### 1. Mismatch related to validation

Confidence in the data, tools, and one's own interpretations of knowledge representations was fundamental to scientists' willingness to engage in and continue engaging in systems biology analysis. Validations occurred from the start during Confirmation and continued throughout the analysis. For Confirmation and Separating the Wheat from Chaff the scientists needed the following data and interactions for their validations: Surrogates for test statistics, such as provenance; counts of interactors, articles, and experiment types associated with an interaction; easy sorting of table fields to locate expected results; and explanations of tool algorithms for processing and integrating data. These features and content were largely present but, at times, were incomplete. Resolving the incompleteness and thus the mismatches for these earlier stages of analysis is not particularly difficult to achieve technologically. Doing so would be a low cost way to enhance scientists' trust early on and possibly instil more tolerance for uncertainty later. It could be that the more confidence scientists gain early on, the more "forgiving" of the tool and data they will be later when dealing with the unavoidable incompleteness and uncertainty of contextualizing biological relationships.

Results show that in later stages ambiguous concepts such as interaction, pathway, and molecule type became increasingly important to scientists' analysis and validation of potentially meaningful relationships. Similarly, the scientists increasingly demanded statistical evidence, evidence that often is not easy to mine and provide technologically or that may be non-existent. The field study suggests that part of these complications to validation and consequent mismatches may be allayed if tools give scientists easy means for bringing in their own data and statistics so that they can integrate and analytically manipulate these data with their query results. The development of more statistical models or presentation of test statistics in which scientists can believe is also needed to reduce tool mismatches with validation needs and practices and to help scientists build confidence.

### 2. Mismatch related to transitions from classifying to model-based reasoning

The importance of helping scientists make the transition from classification to model-based reasoning cannot be overemphasized. Given the high rate of attrition at this stage in the field study, it is clear that support in tools for this transition is as important as facilitating each of the stages itself.

During this transition, scientists made judgments about the value of the displayed knowledge representations for their intended inferential purposes. The scientists were not as concerned as earlier about the literal content of the data. Rather knowledge representations and their value to an analysis required data to be structured in ways that allowed the manipulations the scientists sought to perform to find causal relationships. The scientists needed to see that they could manipulate views by interacting with a single value for an attribute, such as a non-stringed value for molecular function, pathway, or biological process. The scientists also needed to inter-relate select sets of attributes to draw inferences about possible chains of events and effects. Moreover, the scientists needed some indication that they would be able to negotiate the meanings of ambiguous concepts relevant to relationships of interest vital to explanatory inferences, such as interaction type. Finally, they needed knowledge representations that cued them about the connections constituting a protein cluster, the purported strength of the connections, the biological implications, and statistical warrants for the clusters. It was also during the Transition that the scientists checked to see if the tools adequately provided means for interacting with and transforming data in scientists' intended ways.

Mismatches between tools and the cognitive transitions scientists made in the move from lists to explanations may be reduced if views of data and relationships more deliberately direct scientists' attention selectively – for example, to good (biologically meaningful) entry points in complex network associations. Mismatches may also be reduced if tools provide easy means for users to arrange, color code, lay out, and sub-divide/filter networks in biologically meaningful ways. The problem is not rich displays of networks and data per se. Visual analysis research shows that for complex tasks displays rich in data actually succeed in evoking productive selective attention despite their density *if they include *domain knowledge relevant to particular types of questions/reasoning [34]. If presentations provide wrong or incomplete information or insufficiently emphasize relationships relevant to explanations ttention to only surface level data, analytical performance and accuracy for a higher order tasks suffer [34,35]. For networks, this relevance requirement means drawing a user's eye to the high dimensional relationships through color, layout, and groupings.

Additionally, mismatches for scientists' transitions to causal modelling may be diminished if tools provide better cues about conceptual relationships. Choices of layouts can provide such cues, with research in visual cognition showing that when the same connections between nodes are spaced dissimilarly users judge different relationships to be important [36]. Overall, building domain knowledge about relationships into designs- not delivering data elements per se – is crucial for overcoming transition-related mismatches.

### 3. Mismatch related to causal mental modelling

For causal mental modelling, the conceptual relationships that the scientists sought were fluid, dynamic, and not fully formalizable. To uncover them through inference, the scientists expected to rearrange and transform relationships and to view the same data from many perspectives and levels of abstraction. They expected to differentiate normal from abnormal biological behaviors and inferentially distinguish causes from catalysts. In the process, they expected to be able to freely and easily change views of data relationships. Mismatches occurred between expectations and tool support because representations that afforded classification earlier were now insufficient. Moreover, interactivity for data manipulation was not attuned to the explorations required for inference amid ambiguity, incomplete knowledge, and multiple scales. The necessity of this interactivity cannot be overemphasized. User controlled interactivity served as important a function in being able to produce new knowledge and gain deep insights as content.

For example, scientists expected to be able to recursively re-assemble relationships and potential causal associations (spatial transformations) in order to infer possible temporal events. Structuring layouts by regulatory relationships organized by biological region would signal interactions over time and space. Some scientists also spoke of wanting to transform workspaces and data relationships into views of overlaid networks – those of interacting gene products, canonical pathways, and diseases associated with pathways.

These presentations would have evoked biological situational awareness. Scientists knew they could not see actual representations of the dynamics and variability of the genomic and molecular biosystems they sought to analyze. They needed cues from the data and views to infer multi-dimensional chains or loops of actions and outcomes. They wanted certain content to be perceptually highlighted and wanted cues from layouts and groupings about potential biological meanings inherent in visible network configurations. Additionally, side by side graphics (e.g. canonical pathways and a scientist's proteins interactions of interest) would have cued functional associations between previously known and unknown genes.

Other bioinformatics studies have similarly articulated this need for tools to "go beyond simplistic graphical models and mere compliance with accepted standards to provide many different types of network views, each at a different level of abstraction" because relationships among proteins, interaction partners, and interaction types often differ based on a cell state or location [32, 2657]. Specifically, researchers have called for more pathway information, overlays and relationships between molecular interactions and pathways, views of regulatory relationships within pathway contexts, and molecular interactions in relation to many interconnected pathways at once [28,29]. Researchers also have argued that tools need to provide aggregates and details of the same elements, interactivity for selectively expanding neighbors of specified proteins, capabilities for users to specify semantic substrates for layouts, and sub-graphs of network motifs tied to biological concepts [10,29]. Results from the field study corroborate the centrality of these design issues and now show the direct connection between them and scientists' core processes of explanatory reasoning. Field study results also underscore the need to couple these views with adequate interactivity for data and view manipulation and with support for scientists' continuously interleaved validation processes. Without addressing such design improvements, mismatches are likely to continue.

## Conclusion

In the current bioinformatics research literature, models of scientists' flows of analysis for systems biology explorations are incomplete. For design purposes, more comprehensive, empirical models of scientists' higher order cognition are prerequisites for creating tools that effectively and usefully match biomedical researchers' actual ways of knowing and reasoning. In this field study, as in other bioinformatics usability research, scientists could successfully pose and answer "What?" questions with the tools – e.g. What hidden relationships innovatively mined from the literature show unexpected biological relationships? But the scientists – and biomedical researchers in general today – could not adequately achieve new explanatory insights into the how's and why's of biological associations and outcomes within and across biological regions. Given scientists' shared stages of analysis, their multiple complementary modes of reasoning, and their interplay of loose and strict analysis, it is clear why scientists in this study – as in other studies – more easily gained surface level insights than deep insights. Tools strongly support surface level insights by categorizing and organizing data displays in ways that resonate with scientists' approaches to classifying data and judging relationships to be broadly interesting or not. By and large, scientists could perform these tasks through read-offs, and outcomes were motivating. Yet once scientists gained surface insights, they wanted to do more. They wanted to construct credible hypotheses. Findings show that to facilitate deep insights and hypothesizing bioinformatics tools must do a better job of matching scientists' needs and practices. They have to better match scientists' processes for validating throughout analysis, their transition from classification to explanatory mental modelling, and their processes of mental modelling across scales and amid uncertainty.

For design purposes, the field study results reveal the important role that technologies for systems biology analysis play in scientists' exploratory cognition. Because tools are the prime workspaces in which biomedical researchers view and interact with representations of knowledge to further construct and transform knowledge for hypothesizing purposes, technologies shape the course and completeness of this analysis. To varying extents and with qualitative consequences, tools shape scientists' intentions and opportunities for causally exploring known and unknown biological associations according to the domain-driven standards of practice and excellence that they value. As the field study shows, if tools do not match scientists' intentions scientists will likely downgrade their goals, no longer accepting a tool as an aid for formulating an hypothesis. From this perspective, effective translational biomedical research hinges on tool designs matching scientists' higher order cognition as influenced by their domain knowledge and analytical practices.

The results of this field study suggest design objectives and strategies that may reduce mismatches. More research, prototyping, and formative evaluations are needed before requirements can be specified at a fine enough conceptual grain to inform scope (how many tools) and tool designs in a widespread way. Toward this end, field study results suggest that design choices should be informed by objectives and strategies related to: generating trust, reducing search and analysis spaces during classification activities, contextualizing relationships for causal explanations, highlighting and cuing biologically meaningful concepts, and giving users flexible interactivity for core analytical moves and strategies. The Supplemental Material provides more details about these design strategies.

Creating optimal designs that support scientists in integrating the multiple modes of reasoning required for explanatory analysis under uncertainty is the next great frontier in bioinformatics usability. In our national center, NCIBI, the results of this study are now being addressed through many modifications and re-designs to the tools, aimed at overcoming the three mismatches described here.

## Methods

The field study aimed to analyze biomedical researchers' higher order cognitive processes for formulating hypotheses during analyses of protein-protein interactions. Results needed to be framed in a way that could inform tool designs for greater usefulness for higher order reasoning. As in other human-computer interaction studies in bioinformatics [37], qualitative field methods were used to explore scientists' work practices and cognitive processes.

### Participants and their research

Twelve biomedical experimentalists from a large university Medical Center were observed and afterwards interviewed as they analyzed protein-protein interactions and related biological concepts to gain novel insights into disease mechanisms. Two cellular biology experimentalists from the Department of Molecular, Cellular, and Developmental Biology and one biostatistician from the School of Public Health were also observed and interviewed. Table 5 shows the genders and roles of the scientists and the number of times they were observed and interviewed. Four of them worked in pairs, as shown by the groupings in the table. Observations averaged 1.5 hours, totally roughly 31.5 hours of observed sessions. Interviews were conducted after each observation, lasting on average 20 minutes and totalling 8.9 hours all combined.

**Table 5:** Inquiry and observation sessions per scientist

Gender	Role	# Observations	# Interviews
M	Research Scientist	8	9
M	Research Scientist	8	9

M	Professor	2	2
M	Postdoctoral Researcher	1	1

M	Research Scientist	1	1
M	Research Scientist	1	1

F	Research Scientist	1	1
F	Biostatistician	1	1

F	Research Scientist	1	1

F	Research Scientist	1	1

M	Research Scientist	3	5

M	Professor	1	1

M	Professor	1	2

F	Professor	1	1

M	Research Scientist	1	2

		Total = 21	Total = 27

The scientists studied different problems, diseases, and relationships. They proceeded at their own paces and defined their own intentions, analytical tasks, moves and strategies. All the scientists were familiar with and had some experience in systems biology analysis.

### Tools

The tools the scientists used – MiMI, MiMI-Cytoscape, and SAGA (for subgraph matching) – have been described earlier in the Results section. At the time of the field study, the MiMI database was comprised of 117,549 molecules and 256,757 interactions integrated from the ten databases [38]. During the course of the study, MiMI evolved from an XML database to a relational database to facilitate multi-user and arbitrary query functionality. The MiMI database provides extensive data on biological concepts for molecules and interactions, as detailed in Table 6.

**Table 6:** Categories of information

Types of Information	Screen	Types of Information	Screen
Possible Names/Aliases	Molecule	Interaction/Direction	Interaction

Biological Process [GO]	Molecule	Interaction Site	Interaction

Molecular Function [GO]	Molecule	Conditions	Interaction

Cellular Component [GO]	Molecule	Experiments Used	Interaction

Homology	Molecule	Descriptions (from lit)	Both

List of all Interactions	Molecule	Provenance	Both

**Figure 1:**
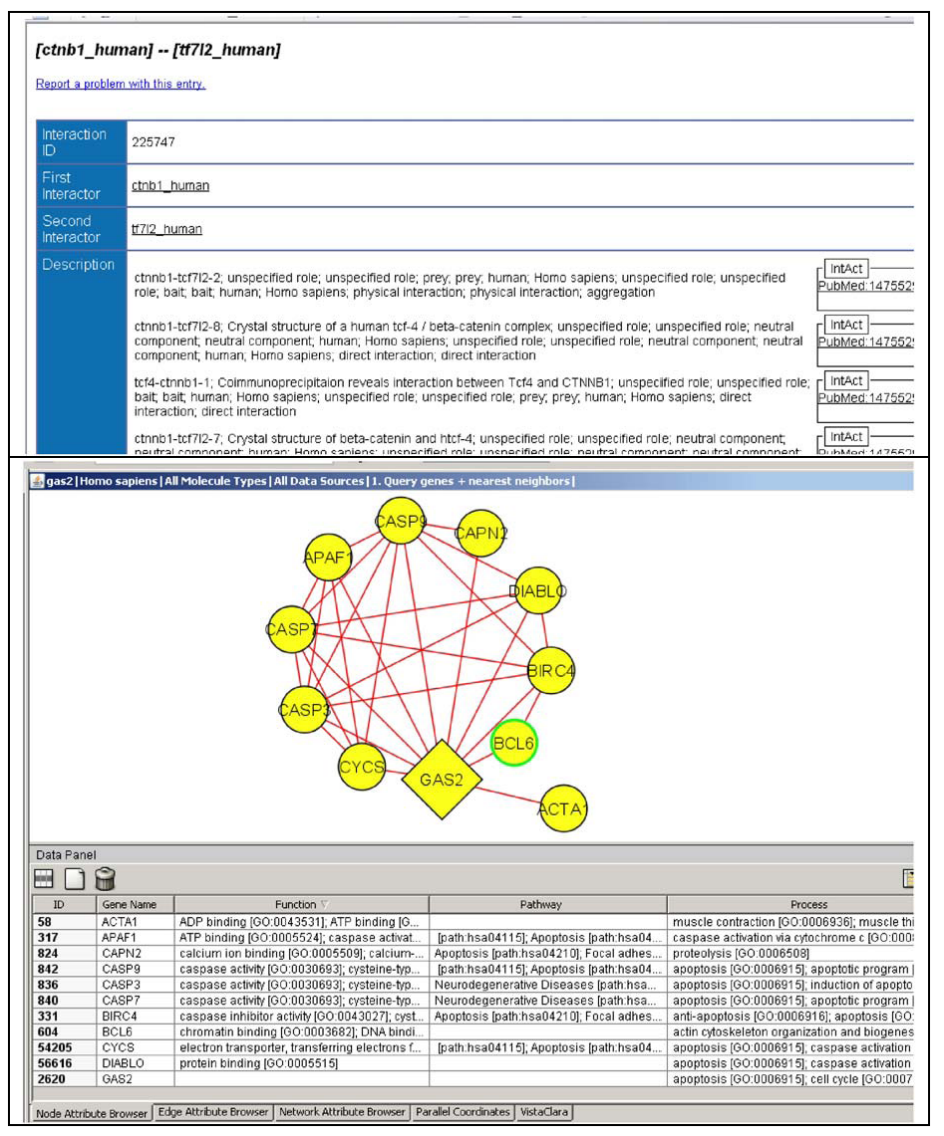
**Screen shots of MiMI and MiMI-Cytoscape**.

In the versions scientists used, three MiMI screens provided query results, which included, respectively: (1) an overview of query results for selecting items of interest, (2) a screen of detailed data on a selected molecule, and (3) a screen of detailed data on a selected interaction (see Figure 1). In the MiMI plug-in for Cytoscape the visualized protein interaction network based on MiMI data and query results was linked to a table displaying details about the graphed genes, gene products, and interactions (see Figure 1). Interacting with the graphics, scientists were able to select data from the network and tabular views, sort tables, lay out networks based on predefined layouts, zoom, pan, and through extensive operations perceptually encode and filter data. They were able to use Cytoscape's color coding (visual mapping) and other available plug-ins, e.g. to see network statistics.

### Data collection

In scientists' naturalistic settings one to two observers took notes with IRB consent. IRB consent assured anonymity to participants, no risk to their work or performance evaluations, and the freedom to end their participation at any time. The scientists performed and thought-aloud during analysis. Sessions were not video- or audio-taped; participants' laboratory spaces were often too small and noisy. Scientists' processes for entering queries were not studied.

The two observers and interviewers were experienced and skilled in taking notes rapidly. During observations, they captured the following data: Verbatim (or close to verbatim) think-aloud comments/questions; times at the start and end of various sub-tasks; and observed actions, outcomes and impasses. Observers recorded missteps – those the scientists noted and those they did not. In think-aloud comments, scientists typically conveyed and observers captured various task objectives, intentions, deliberations, lines of reasoning, confusions, confirmations, judgments about emerging findings, reactions to impasses, and satisfaction. Observers did not interrupt scientists as they worked nor did they guide scientists toward "correct usages" of tools or tool features.

Non-structured interviews after observations sessions focused on scientists' cognition and needs for support from the tools but were tailored to the specific observations, research problem, and analytical objectives of each scientist. Interviews asked scientists about their tasks, their reasoning behind task choices and behaviors, and their ordering and combining of tasks. Interview notes recorded scientists' responses verbatim – or close to verbatim – but did not similarly capture the questions posed by the interviewer, since he or she often doubled as the note-taker. Interview notes predominantly documented as best the interviewers could the exact language of verbal exchanges, sporadically adding impressionist notes that highlighted a theme or pattern that seemed relevant to the study. Some scientists participated in additional interviews that involved follow up questions about a scientist's research project and methods.

### Data analysis

Using qualitative methods for grounded theory analysis adapted to design ends [39], notes of observations and interviews were read holistically several times and then critically analyzed. Data analysis related verbatim/near verbatim think-alouds and interview responses to the observed task behaviors, task durations, and impressions from observers' notes. For each case, narrative and procedural scenarios were constructed, capturing the scientist's analysis for his or her research problem. When pairs of scientists worked collaboratively, the observation of both individuals was treated as a single case scenario. For cases of analysis involving multiple observations, one unified scenario was constructed for the problem under study. Denser data due to multiple observations and/or interviews were not given any more weight. These cases typically just filled in details otherwise missing about reasoning, scientists' flow of analysis, and composite stages and tasks. Formal content coding was not applied because the data analysis objective was not so much to thematically or sociolinguistically characterize users and their work or to theorize about higher order scientific cognition per se. Rather the objective was to abstract context-driven patterns of thinking and behaviour that could inform design. Results from overly detailed coding typically do not translate readily into design.

Analysis was informed by theories of higher order cognition for complex analysis and visual analytics rather than by cognitive models for low level procedures, which are typically the unit of study in cognitive task or hierarchical task analysis. Other theories relevant to computer-supported complex tasks also framed analysis but not as pre-ordained conclusions. Relevant socio-technical conceptual frameworks from human-computer interaction, design, and visual cognition afforded looking at the data from multiple perspectives.

Outcomes were related to the analytical gaps in visualization tools identified in [26]. Items scientists saved or mentioned wanting to save in each case were identified to indicate if scientists placed enough value on outcomes and insights to store them for later personal reference, sharing, or both. Variations across cases were identified and explained. From this analysis, a user model organized around dominant cognitive processes in various stages of analysis was developed.

## Competing interests

The author declares that they have no competing interests.

## Supplementary Material

Additional file 1 Mirel_SupportCogSysBiology_SuppMatl. A. pdf file providing details of design implications to overcome each mismatch. Click here for file
