# Supplementary material for: Supporting cognition in systems biology analysis: findings on users' processes and design implications
Source: J Biomed Discov Collab. 2009 Feb 13;4:2. doi: 10.1186/1747-5333-4-2 (PMC2649900; doi:10.1186/1747-5333-4-2)
Supplement: Additional file 1 — Mirel_SupportCogSysBiology_SuppMatl. A. pdf file providing details of design implications to overcome each mismatch. [file 1747-5333-4-2-S1.pdf]

Supplemental Material

Design Implications

Results from the field study imply a number of design strategies proposed below. Some strategies give citations to other relevant work that appears in the research literature, with reference details provided at the end. Overall guidance for visualization design can be found in Uetz et al, 2005 and Schrinivasan and van Dijk, 2008.

Design strategies are organized by the three mismatches discussed in the article and by the user needs and questions that are prominent in each mismatch. Sometimes design strategies may satisfy more than one mismatch. The benefit of this organization is that it reveals the design strategies that need to be implemented together, not as discrete pieces, in order to overcome mismatches.

Mismatch 1. Designing to Better fit Validation Needs

A.

**User Need**	**Can I trust the tool?**<br>**Can I trust statistics that the tool generates (e.g. enrichment scores, cluster co-efficients?)**
**Mismatch**	1
**Objective**	Generate trust
**Design strategies**	• Reveal underlying processing logic<br>• Reveal logic for generating statistical measures.<br>• Designate data are missing<br>• Provide "why?" provenance and semantics of data integration (Chapman et al, 2008)

B.

**User Need**	**Can I trust the reliability and credibility of the data?**
**Mismatch**	1, 2
**Objective**	Generate trust
**Design strategies**	Provide test statistics and surrogates for credibility, e.g:<br>• # of evidences/types experiments<br>• Types of experiments<br>• # of articles citing gene/ interactions<br>• Dates of articles & links to them<br>• # of interactions a gene has<br>• Literature extracts from natural language processing (NLP)

C.

**User Need**	**Can I narrow in from the start on specific data and properties of interest for exploration and get to just that literature for verification?**<br>**Are there discrepancies between displays and what I know conceptually?**
**Mismatch**	1, 2
**Objective**	Generate trust, Reduce search /analysis space, Contextualize and cue biological relationships
**Design**	Provide form-based queries informed by filters that represent the most important

**User Need**	**Can I narrow in from the start on specific data and properties of interest for exploration and get to just that literature for verification?**<br>**Are there discrepancies between displays and what I know conceptually?**
**Strategies**	criteria that scientists use to find relationships of interest (those mentioned in Mismatch 2) (Jayapandian and Jagadish, 2008)<br>Enable scientists to interact with multiple-scale views of complex query results.

D

**User Need**	**How confident am I that displayed interactions that I'm interpreting are not happening by chance alone?**
**Mismatch**	1, 2
**Objective**	Generate trust, Reduce search/ analysis space, Contextualize and cue biological relationships, Give users flexible interactivity
**Design strategies**	Develop/let users run algorithms for significance testing on overall network structures and motifs (Barabasi and Oltvai, 2004)<br>Reveal the logic and parameters of the algorithms<br>Offer additional indicators of the strength of relationships – e.g. term enrichment statistics(MeSH, GO); and reveal the logic of these computations<br>Read in and allow color coding and aggregation on regulatory/expression values

Mismatch 2. Designing to Better fit the Transition from Classifying to Mental Modelling

**User Need**	**Will I be able to move from static multidimensional relationships of interest to dynamics of association and effect?**
**Mismatch**	2
**Objective**	Generate trust, Reduce search/ analysis space, Contextualize and cue biological relationships, Give users flexible interactivity
**Design Strategies**	Clarify/display what comprises an interaction and a molecule<br>Additionally, give prominence to other high priority information for seeing patterns and relationships: GO annotations, homology, pathways, reactions, interrelated layers of GO annotations across classes (Dadzie and Burger, 2005)<br>Provide the ability to perceptually encode on node and edge traits, including counts or types of experiments that showed a particular interaction (Barsky et al, 2007)<br>Highlight indirect interactions and combine visual highlighting and motion to draw selective attention to neighbors and sub-graphs, especially motifs, or clusters sharing attributes. (Ware and Bobrow, 2002)<br>Provide several side by side views for diverse perspectives on biological relationships have them dynamically linked on such operations as selection, filtering, and color coding<br>In views rich in conceptual biology data, cue groupings, scales, and content relevant to domain-based inferences (Baldonado et al, 2000)<br>Show/provide the ability to import one's own data (Cline et al, 2007)<br>Show/provide abilities for users to: Aggregate by self-specified fields and select

User Need	**Will I be able to move from static multidimensional relationships of interest to dynamics of association and effect?**
	values; Quickly perceptually encode to show additional dimensions; View 1+ canonical pathways that are interactive

3, Designing for a Better fit with Explanatory Mental Modeling and Causal Inferences

A.

User Need	**What entities are interacting?**<br>**Which interactions are part of regular behavior for stability and normal functioning?**<br>**Which interactions are aberrations associated with disease?**
**Mismatch**	3
**Objective**	Contextualize and cue biological relationships
**Design strategies**	Represent and provide ways to perceptually encode (e.g. color code) on molecular attributes and display the encoding on pathways (Efroni et al, 2007)<br>Provide capabilities for user annotations (publicly shared if desired) and give some means of standardizing them to afford perceptual encoding, filtering, etc. (Chen et al, 2008)<br>Represent sub-networks associated with disease (Chuang et al, 2007)

B.

User Need	**Can I inventively group genes and relationships of interest to infer causal relationships**?
**Mismatch**	3
**Objective**	Trust, Reduce search/ analysis space, Contextualize and cue biological relationships
**Design strategies**	Represent relationships across GO classes and hierarchical levels (e.g. a specific function "is involved in" a certain process and "acts in" a certain component; or provide visual pivots to show many hierarchical levels in multiple classes and cross-membership) (Myre et al, 2006; Robertson et al, 2002)<br>Provide capabilities for users to group clusters of interactions into aggregates defined by a superordinate class (e.g. GO category, perhaps at a certain level of the hierarchy). Provide the ability for users to create "smart" aggregates on available traits (Tesone and Goodall, 2007)<br>Represent relationships between gene/protein interactions and significantly enriched MeSH terms, letting users select the relationships to display<br>Highlight motifs in biological networks with the significance of their frequency and let users interactively impose biological traits on visualized motifs to find biological meaning, (Schrieiber and Schwobbermeyer, 2005)<br>Provide zoom capabilities that leave context visible<br>Provide the ability to drill down into aggregates and roll up again<br>Reveal computations on which a tool's pre-calculated clusters are based.<br>Build in hierarchical graph structures (in which nodes contain graphs) to accommodate displays of aggregates

C.

User Need	**Can I place interactions in context to infer and judge the credibility of variable behaviors, contingencies, and dynamic effects?**
**Mismatch**	3
**Objective**	Trust, Reduce search/ analysis space, Contextualize and cue biological relationships
**Design strategies**	Provide views/layouts of gene interactions that suggest temporal contexts , e.g. gene interactions layered by regulatory processes (Barsky et al, 2007)<br>Represent (e.g. through overlays) gene/protein interactions and states in relation to canonical pathways, in relation to "disease-ome," in relation to regulatory relationships, or in relation to all three (Reese et al, 2005; Efroni et al, 2007)<br>In representations of neighbors, draw attention/let users draw attention to biologically meaningful neighbors, chains/loops of interactions, or factors limiting behaviors in a hypothetical biological event<br>Provide graph-theoretic statistics on networks with cues to implications for inferring biological meaning and with significance values to judge the likelihood of a topological structure occurring by chance alone in the database (Zhang et al, 2007; Bader and Hogue, 2003; Wong et al, 2006)<br>Provide visual indicators signaling confidence levels when statistical values are encoded by color, size, thickness (Holloway et al, 2008)

D.

User need	**Can I spatially transform my mental model of causal relationships to better develop and validate explanations of biological events and consequences?**
**Mismatch**	3
**Objective**	Reduce search/analysis space, Contextualize and cue biological relationships, Give users flexible interactivity
**Design strategies**	Build in functionality for graph customizations, e.g. let users construct a: workspace for side by side comparisons (Jonker et al, 2005; Kerpedjiev and Roth, 2001)<br>Build in functionality for aggregating data, including custom aggregations and for perceptually encoding by aggregates.

References for the Supplemental Material

Bader G, Hogue C: **An automated method for finding molecular complexes in large protein interaction networks.** *BMC Bioinformatics* 2003, 4:2-29.

Baldonado M, Woodruff A, Kuchinsky A: **Guidelines for using multiple views in information visualization**. *Proceedings of the Working Conference on Advanced Visual Interfaces (AVI '00)* New York: ACM Press; 2000:110-119.

Barabasi A-L, Oltva, ZN: **Network biology: understanding the cell's functional organization**. *Nature Reviews Genetics* 2004, 5:101-113.

Barsky A, Gardy J, Hancock R and Munzner T: **Cerebral: A cytoscape plugin for layout of and interaction with biological networks using subcellular localization annotation**. *Bioinformatics* 2007, 23:1040-1042.

Chapman A, Jagadish HV, Ramanan P: **Effective provenance storage**. *Proceedings of the 2008 ACM SIGMOD International Conference on Management of Data*. Edited by Shasha D, Wang J: ACM Press; 2008, 993-1006.

Chen W-B, Li U, Soong SJ, Chen D: **A guided approach for personalized information search and visualization**. *Interdisciplinary Journal of Information, Knowledge, and Management* 2008, 3:11-20.

Chuang HY, Lee E., Liu YT, Lee D, Ideker T: **Network-based classification of breast cancer metastasis**. *Molecular Systems Biology* 2007, 3:140.

Cline M, Smoot M, Cerami E et al: **Integration of biological networks and gene expression data using Cytoscape**. *Nature Protocols* 2007, 2: 2382.

Dadzie A-S, Burger A: **Providing visualization support for the analysis of anatomy ontology data**. *BMC Bioinformatics* 2005, 6:74.

Efroni S, Schaefe, C, Buetow K: **Identification of key processes underlying cancer phenotypes using biologic pathway analysis.** *PLoS ONE*, 2007, 2:e425.

Holloway, D, Kon, M, Delisi, C: **Classifying transcription factor targets and discovering relevant biological features**. *Biology Direct*, 2008, 3:22.

Jayapandian M, Jagadish HV: **Expressive query specification through form customization**. *Proceedings of the 11th International Conference on Extending Database Technology: Advances in Database Technology*: ACM Press; 2008:416-427.

Jonker D, Wright W, Schroh D, Proulx P, Cort B: **Information triage with TRIST**. *Proceedings of 2005 International Conference on Intelligence Analysis* 2005: US Government and Mitre Corporation Last accessed on January 7, 2009 at: https://analysis.mitre.org/proceedings/Final_Papers_Files/22_Camera_Ready_Paper.pdf

Kerpedjiev S, Roth S: **Mapping communicative goals in conceptual tasks to generate graphics in discourse**. *Knowledge Based Systems* 2001, 14:93-102.

Myre S, Tveit H, Mollestad T, Laegreid A: **Additional gene ontology structure for improved biological reasoning**. *Bioinformatics* 2006, 22:2020-2027.

Reese D, Avila-Campilio I, Thorsson V, Schwikowski B, Galitski T: **Tools enabling the elucidation of molecular pathways active in human disease**. *BMC Bioinformatics* 2005, 6:154.

Robertson G, Cameron K, Czerwinski M, Robbins D: **Polyarchy visualization: visualizing multiple intersecting hierarchies**. *Proceedings of the SIGCHI Conference on Human Factors in Computing:* ACM Press; 2002: 432-430.

Schrieiber F, Schwobbermeyer **MAVisto: a tool for the exploration of network motifs.** *Bioinformatics* 2005, 21: 3572-3574.

Schrinivasin Y, Van Wijk J: **Supporting the analytical process with visualizations**. *Proceedings of the 26th Annual SIGCHI Conference on Human Factors in Computing 2008*: ACM Press; 2008:1237-1246.

Shneiderman B. Aris *A: Network visualization by semantic substrates*. Technical Report HCIL-2006-19: College Park, MD: University of Maryland, 2006.

Soon-Hyung Y, Oltvai ZN, Barabasi A-L: **Functional and topological characterization of protein interaction networks**. *Proteomics* 2004, 4:928-942.

Tesone D, Goodall J: **Balancing interactive data management of massive data with situational awareness through smart aggreg/ation**. *IEEE Symposium on Visual Analytics Science and Technology (VAST)* 2007: 67-74

Uetz P, Ideker T, Schwikowski B: **Visualization and integration of protein-protein interactions.** In *The Study of Protein-Protein Interactions- An Advanced Manual.* Edited by Golemis E: Woodbury,NY: Cold Spring Harbor Laboratory Press, 2005.

Ware C, Bobrow R: **Supporting visual queries on medium sized node-link diagrams**. *Information Visualization* 2005, 4:49-58.

Wong PC, Foote H, Chin Jr. G, Mackey P, Perrine K: **Graph signatures for visual analytics.** *IEEE Transactions on Visualization and Computer Graphics* 2006, 12:1399-1413.

Zhang S, Jin G, Zhang X-S, Chen L: **Discovering functions and revealing mechanisms at molecular level from biological networks**. *Proteomics* 2007, 7:2856-2869.
